# Anatomical and Functional Outcomes of Vitrectomy with/without Intravitreal Methotrexate Infusion for Management of Proliferative Vitreoretinopathy Secondary to Rhegmatogenous Retinal Detachment

**DOI:** 10.1155/2021/3648134

**Published:** 2021-07-20

**Authors:** Samir El Baha, Mahmoud Leila, Ahmed Amr, Mohamed M. A. Lolah

**Affiliations:** ^1^Department of Ophthalmology, Alexandria University, Alexandria, Egypt; ^2^El Baha Eye Center, Alexandria, Egypt; ^3^Retina Department, Research Institute of Ophthalmology, Giza, Egypt

## Abstract

**Purpose:**

To assess the anatomical and functional outcomes of intravitreal infusion of methotrexate (MTX) during pars plana vitrectomy (PPV) for proliferative vitreoretinopathy (PVR) associated with rhegmatogenous retinal detachment (RRD).

**Methods:**

Comparative interventional nonrandomized study including consecutive patients who had vitrectomy for RRD. The study included six groups. Groups I (established PVR), II (high risk of PVR), and III (no risk of PVR) comprised prospectively recruited study eyes, which received PPV and adjuvant intravitreal MTX infusion equivalent to 400 *μ*g/0.1 mL. Groups IA, IIA, and IIIA comprised retrospectively recruited control groups. Main outcome measures were retinal reattachment at the end of 6 months, visual outcome, and complications. Chi-square test or Fisher's exact test analyzed categorical variables. ANOVA test and Kruskal–Wallis test analyzed quantitative variables. Mann–Whitney *U*-test and independent *t*-test evaluated the difference between each group and its control. Comparison between two paired groups was done by Wilcoxon Rank test. The Kaplan–Meier method was used for survival analysis and the log-rank test estimated differences in event-free survival across the groups. *P* was significant at <0.05.

**Results:**

The study included 190 eyes of 188 patients. Study Groups I, II, and III included 42, 35, and 24 eyes, respectively. Mean age was 45 years. Male gender constituted 70% of patients. Mean follow-up period was 6 months. Control Groups IA, IIA, and IIIA included 30, 30, and 29 eyes, respectively. Mean age was 50 years. Male gender constituted 50%. Mean follow-up period was 7 months. Median rate of retinal reattachment was 82% in the study eyes versus 86% in the control eyes. The difference in the retinal reattachment rates between each study group and its respective control was not statistically significant, Group I-IA (*p*= 0.2), Group II-IIA (*p*=0.07), and Group III-IIIA (*p*=0.07). BCVA improved by a mean of 4 lines in the study eyes versus 3 lines in the control eyes. The difference in visual outcome between each study group and its respective control was statistically significant between Groups II-IIA and III-IIIA, *p*=0.03, but not between Groups I-IA, *p*=0.07. We did not detect complications attributed to MTX use in the study eyes.

**Conclusion:**

Intravitreal infusion of MTX during PPV is a safe adjuvant therapy in RRD patients with and without PVR. MTX yields superior functional outcomes in patients at high risk of PVR and in patients with no risk of PVR compared to PPV without MTX, but not in cases with established PVR. MTX did not confer an additional advantage in terms of retinal reattachment rate. *Summary*. Proliferative vitreoretinopathy is a major cause of failure in surgery for rhegmatogenous retinal detachment. Methotrexate as an adjuvant therapy blocks essential drivers in the pathogenetic cascade leading to PVR. Intravitreal infusion has the advantage of blocking the pathology in its nascence and obviates the need for repeated intravitreal injections of the drug.

## 1. Introduction

Proliferative vitreoretinopathy (PVR) represents a robust wound-healing response of the retina to injury produced by retinal detachment. The retinal cellular elements involved in this response are legion, and they work in tandem in a multipronged cascade that eventually establishes PVR. The pathogenetic process is based on three factors that are considered the hallmark of PVR. Firstly, migration of retinal pigment epithelial (RPE) cells and cytokine-producing immune cells through the retinal break(s) and dehisced blood-retina barrier (BRB), respectively, along with activation of retinal astrocytes and Müller cells. Secondly, inflammatory cytokines trigger metaplasia of RPE cells into myocontractile cells and proliferation of retinal glial elements. Finally, these cells produce an extracellular matrix and undergo relentless fibrocellular proliferation in the vitreous and along both sides of the retina with the formation of contractile membranes [1–6]. PVR is considered the most implacable complication of retinal detachment that claims 75% of failed retinal detachment surgical repair [7, 8]. Currently, the only treatment of PVR is surgical removal of periretinal membranes, although the functional outcome of surgery is far from satisfactory. Mean percentage of patients gaining ambulatory vision (≥5/200) varies widely from 35.5% to 85% of successful retinal reattachment cases [9–12]. The presence of inflammatory progenitors, the proliferative nature of the disease, and the unsatisfactory functional outcome of PVR surgery catalyzed the hypothesis that antineoplastic drugs used as pharmacologic adjuvants during pars plana vitrectomy (PPV) could halt the sequence of events leading to PVR [5, 7, 8, 13–20]. Methotrexate (MTX) is a folate analogue that inhibits cell proliferation through competitive inhibition of enzymes requiring folate. These enzymes are essential for deoxyribonucleic acid (DNA) and ribonucleic acid (RNA) synthesis [21]. At an intraocular dose of ≤400 *μ*g/0.1 mL, MTX inhibits cytokine-producing immune cells and cellular proliferation; however, it has no effect on cellular migration. Thus, it can effectively neutralize two major components of the pathologic sequence leading to PVR, namely, induction of RPE metaplasia and proliferation of myocontractile cells and glial elements of the retina [13, 14, 21]. Nevertheless, the relatively short therapeutic half-life of MTX when delivered intravitreal is a significant shortcoming when considering the protracted time span of PVR process since nascence through contractile membrane formation. Müller cell activation starts almost immediately; whereas cellular proliferation starts as early as the 4th day after the onset of retinal detachment, and the disease course continues for at least 90 days [6, 7, 16]. Since the therapeutic half-life of MTX inside the vitreous cavity is only 3 to 5 days; therefore, multiple injections are required to suppress the PVR process during that period [22, 23]. In comparison, intravitreal infusion of MTX during PPV has been reported to suppress PVR effectively. The rationale for this route is based on the easy penetrance of the low-molecular weight MTX into the retinal tissues, and hence, the achievement of a stable tissue concentration that produces a uniform dosing of the drug as opposed to a single bolus delivered at the end of surgery [7]. The aim of this study is to assess the anatomical and functional outcomes of intravitreal infusion of MTX during PPV for PVR associated with retinal detachment.

## 2. Patients and Methods

This is an interventional comparative nonrandomized case series conducted in a retina tertiary care center between February 2019 and January 2021. The study included all consecutive patients who had PPV for rhegmatogenous retinal detachment (RRD). The study included six groups. Groups I, II, and III comprised prospectively recruited study eyes that received PPV and adjuvant intravitreal MTX infusion equivalent to 400 *μ*g/0.1 mL. Group I (established PVR) included eyes with RRD and PVR C located posterior (CP) or anterior (CA) to the equator and involved 1 or more clock hours. Group II (high risk of PVR) included eyes with recent-onset RRD ≤ 1-week duration and no clinical signs of PVR but with one or more risk factors for developing PVR. Group III (no risk of PVR) included eyes with recent-onset RRD ≤ 1-week duration and no clinical signs of PVR or co-exiting risk factors for developing PVR. Groups IA, IIA, and IIIA comprised retrospectively recruited control eyes with established PVR, high risk of PVR, and no risk of PVR, respectively. Patients in these groups had PPV without adjuvant intravitreal MTX infusion. Risk factors for developing PVR were identified as aphakia, high myopia, vitreous hemorrhage, hypotony, suprachoroidal effusion/hemorrhage, giant retinal tear, RRD involving ≥2 quadrants, penetrating trauma with or without retained intraocular foreign body (IOFB), recurrent retinal detachment after previous surgery, intraoperative cryotherapy, and retinotomy or relaxing retinectomy. Staging of PVR followed the guidelines of the Retina Society classification of PVR of 1983 and the updated classification of retinal detachment with PVR of 1991 [24, 25]. Exclusion criteria included patients <18 years old, pregnant and breast-feeding mothers, co-existing pathology that might induce PVR such as proliferative diabetic retinopathy (PDR) or uveitis, co-existing congenital anomalies or hereditary vitreoretinopathies, and patients who were unable to complete at least 6 months of follow-up. Main outcome measures were successful reattachment of the retina at the end of 6 months, with removal of silicone oil or absorption of gas tamponade and without additional surgery, visual outcome, and complications of MTX use. Patients who presented with retinal redetachment after primary PPV underwent repeat surgery within 3 weeks. They were not included in successfully reattached cases even if that was achieved after additional surgery. All recruited patients received full ophthalmological assessment including history taking, best-corrected visual acuity (BCVA) using Snellen's decimal notation, anterior segment slit-lamp examination including applanation tonometry, indirect ophthalmoscopy with 360° scleral indentation and slit-lamp biomicroscopy.

### 2.1. Surgical Technique

All surgical procedures described herein were performed by a single retina surgeon (S.B.). Surgical technique consisted of a standard 3-port 23-gauge PPV. Patients with co-existing cataract that was dense enough to impede visualization during PPV or in whom sparing a clear crystalline lens would hinder elimination of proliferative membranes underwent phacoemulsification with implantation of a posterior chamber intraocular lens within the capsular bag before starting PPV. In the study groups, MTX infusion was prepared from a commercially available MTX vial (50 mg/2 mL). A volume of MTX equivalent to 40 mg/mL was withdrawn and added to a 500 mL balanced salt saline (BSS) bottle at the start of the infusion line. This would achieve an intraocular concentration equivalent to that of a 400 *μ*g intravitreal MTX injection, given that the volume of the human eye is approximately 5 mL. In the control groups, the infusion line contained pure BSS. The time duration of all surgeries did not exceed 60 minutes. The surgical procedure consisted of core vitrectomy followed by injection of triamcinolone acetonide to help identify the posterior hyaloid. If not already induced, PVD was performed by applying active suction at the edge of the ONH. Once induced PVD was continued as far anteriorly as possible. That was followed by shaving of the vitreous base. The surgeon selected as per his discretion among surgical maneuvers such as cryotherapy, endodiathermy, endolaser, application of scleral band, peeling of epiretinal membranes and/or internal limiting membrane (ILM), removal of subretinal membranes through retinotomy, relaxing retinectomy, use of perfluorocarbon liquid (PFCL), and choice of type of intraocular tamponade. Finally fluid/air exchange was performed followed by air/silicone oil or air/gas exchange. All patients with successful retinal reattachment and no evidence of recurrent retinal proliferation, who received silicone oil tamponade had a second surgery for silicone oil removal 3 months after the initial surgery. Postoperatively, patients were examined at 1-day, 1-week, 1-month, and 3-monthly thereafter.

### 2.2. Statistical Analysis

Data were described by means, standard deviation and frequency, percentages for quantitative and qualitative variables, respectively. Categorical variables were analyzed by the Chi-square test or Fisher's exact test, while differences in quantitative variables between the 3 groups were analyzed by the one-way ANOVA test for normally distributed variables and the Kruskal–Wallis test for nonnormally distributed ones. Differences between each group and its controls were tested by the Mann–Whitney *U*-test for nonparametric data and by the independent *t*-test for parametric ones. Wilcoxon Rank test was used to compare between two paired groups regarding quantitative data, and nonparametric distribution was done using the Wilcoxon Rank test. Survival analysis was done by the Kaplan–Meier method to estimate the event-free survival, where the event was defined as recurrence of retinal detachment. Differences in event-free survival across the groups were evaluated by the log-rank test. *P* value < 0.05 was considered significant.

#### 2.2.1. Statistical Power

A two-sided log-rank test with an overall sample size of 190 subjects (89 in the control group and 101 in the treatment group) achieves 80.0% power at a 0.050 significance level to detect a hazard ratio of 1.96 when the proportion surviving in the control group is 0.8950 with a difference in survival of 9%. The study lasts for 24 time periods, of which subject accrual (entry) occurs in the first 13 time periods.

## 3. Results

### 3.1. Characteristics of the Study Population (Shown in Table 1)

The study included 190 eyes of 188 patients, of which 101 eyes of 99 patients comprised the study groups. Groups I, II, and III included 42, 35, and 24 eyes, respectively. Mean age was 45 years (range: 18-71; SD: 15). Male gender constituted 70% of patients. Mean follow-up period was 6 months (range: 6-8; SD: 0.3). In Group II, recurrent RRD after previous surgery was the main risk factor for PVR (60%), followed by penetrating trauma (11%), vitreous hemorrhage (11%), giant retinal tear (8.5%), and suprachoroidal hemorrhage (8.5%). There was no statistically significant difference between the 3 groups in terms of mean values of gender, age, status of the crystalline lens, baseline BCVA, or follow-up period. Silicone oil tamponade was used in 91% of the overall sample, and in 95%, 83%, and 96% of Groups I, II, and III, respectively. Groups IA, IIA, and IIIA included 30, 30, and 29 eyes, respectively. Mean age was 50 years (range: 20-76; SD: 13). Male gender constituted 50%. Mean follow-up period was 7 months (range: 6-12; SD: 1.7). In Group IIA intraoperative use of cryotherapy was the main risk factor for PVR (93%), followed by high myopia (13%). Silicone oil tamponade was used in 86% of the overall sample, and in 90%, 78%, and 89% of Groups IA, IIA, and IIIA, respectively. Statistically significant differences were present between Groups I and IA in the status of the crystalline lens (*p*=0.01) and follow-up period (*p*=0.003), between Groups II and IIA in the follow-up period (*p* ≤ 0.001) and between Groups III and IIIA in gender distribution (*p*=0.001).

### 3.2. Anatomical and Functional Outcomes (Shown in Tables 2to 5)

#### 3.2.1. Anatomical Outcome

In the study eyes, we achieved a successful retinal reattachment rate after a single procedure in 74%, 77%, and 96% in Groups I, II, and III, respectively (*p*=0.08), with a median rate of retinal reattachment 82% of the overall study sample. We did not detect complications in any group attributed to MTX use. Six-month survival analysis was 100%, 59%, and 52% in Groups I, II, and III, respectively (*p*=0.009). In the control eyes, we achieved a successful retinal reattachment rate after a single procedure in 87%, 93%, and 79% in Groups IA, IIA, and IIIA, respectively, with a median rate of retinal reattachment 86% of the overall control sample. The difference in the retinal reattachment rates between each study group and its respective control was not statistically significant, *p* = 0.2, 0.07, and 0.07. Six-month survival analysis across the study and control eyes revealed a statistically significant difference in Groups II-IIA, 59% versus 92.5%, respectively, *p* = 0.003. Neither the status of the crystalline lens in the study and the control groups, nor the number of previous recurrences in Groups I–IA and II–IIA were significant contributing factors in the anatomical outcome described herein.

**Figure 1:**
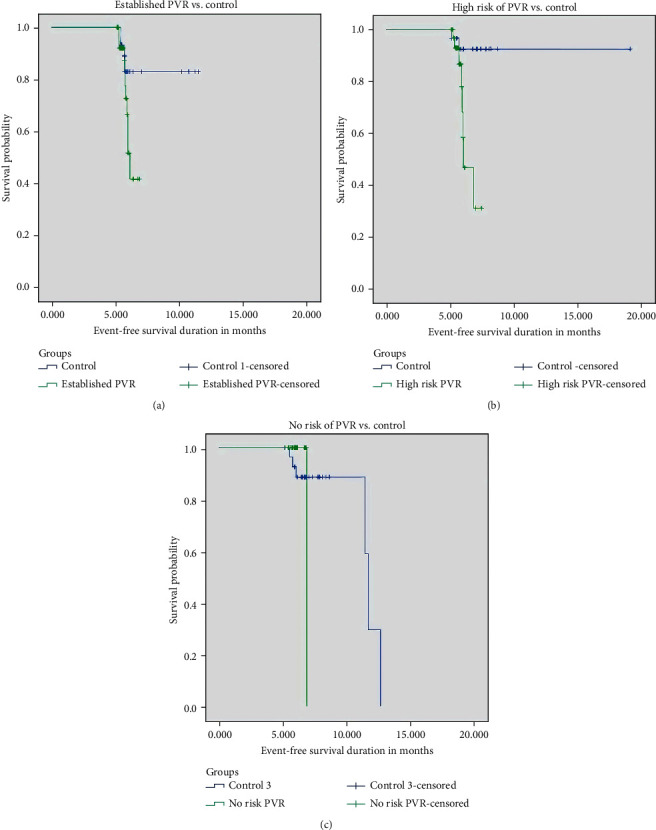
a-c. Six-month Kaplan–Meier survival curve for the study groups and their respective controls. Event refers to retinal redetachment within 6 months postoperatively.

**Figure 2:**
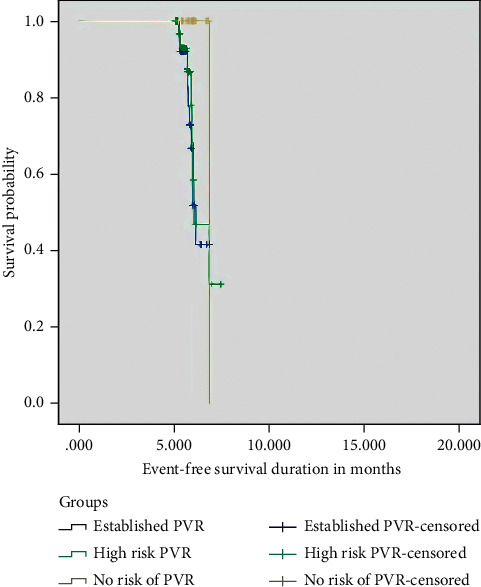
Event-free survival across the study groups.

*(1) Survival Analysis in the Study and Control Eyes. (Shown in Figures 1(a)–1(c) and 2)*. Six-month survival was 52%, 59%, and 100% in Groups I, II, and III, respectively, *p*=0.009. The differences in 6-month survival between Groups I-IA, II-IIA, and III-IIIA were 52% versus 83% (*p*=0.07), 59% versus 92.5% (*p*=0.003), and 100% versus 92.6% (*p*=0.8), respectively.

#### 3.2.2. Functional Outcome

In the study eyes, BCVA improved by 5 lines, 4 lines, and 3 lines in Groups I, II, and III, respectively (*p* = 0.05). Mean improvement of BCVA was 4 lines in the overall study sample. In the control eyes, BCVA improved by 2 lines, 3 lines, and 4 lines in Groups IA, IIA, and IIIA, respectively. Mean improvement of BCVA was 3 lines in the overall control sample. The difference in visual outcome between each study group and its respective control was statistically significant between Groups II-IIA and III-IIIA, *p* = 0.03, but not between Groups I-IA (*p* = 0.07).

## 4. Discussion

This study assessed the efficacy of MTX infusion during PPV as a pre-emptive measure in RRD without PVR or at high risk for developing PVR and as a therapeutic adjuvant in established PVR cases. The median overall retinal reattachment rates in the study and control groups were 82% versus 86%, respectively. These rates were not statistically significant. All rates were reported after silicone oil removal or absorption of gas. In terms of MTX use, our retinal reattachment rates in Groups I and II were superior to those reported after a single surgery by De Silva et al. [9] (68%), Lewis et al. [11] (68%), Silicone study group [12] (67% and 68.5%), Asaria et al. [18] (71.2%), Grigoropoulos et al. [26] (51%), and Charteris et al. [27] (51%; placebo arm of a series of cases with RRD and established PVR grade C). In contrast, higher retinal reattachment rates were reported by Lewis et al. [10] (81%), Lam et al. [28] (81.6%), and Wickham et al. [29] (86.8%; the placebo arm of a series of unselected cases with RRD). The latter authors mentioned that 86% of their patients did not have PVR at presentation. Review of studies on PPV for PVR is shown in Table 6. Our retinal reattachment rate after a single procedure in Group III matched the maximum success rate published in the literature (71%-96%) [30]. Most published data on the use of MTX in RRD are derived from retrospective studies [7, 8, 15], pilot studies [16], or small prospective case series [17]. Benner et al. [8] reported retinal reattachment in all 5 eyes with severe PVR using MTX injection as an adjuvant to extended perfluorocarbon tamponade. The authors delivered 12 injections of MTX in one patient and 5 injections in the remaining 4 patients. Nourinia et al. [16] reported 100% retinal reattachment in a series of 11 eyes with PVR grade C using multiple intrasilicone injections of MTX. However, the authors did not remove silicone oil in more than 80% of their cases. Falavarjani et al. [17] reported retinal reattachment in 95.5% of 22 eyes with PVR grade C that received a single intrasilicone oil injection of MTX. The authors reported no statistically significant difference in rates of retinal redetachment between treatment and control arms. Sadaka et al. [7] used MTX infusion during PPV in an uncontrolled retrospective series of 29 eyes with established PVR and eyes at high risk for developing PVR. The authors reported a retinal reattachment rate of 90%. Review of studies on MTX use for PVR is shown in Table 7. To our knowledge, this study is the largest prospective controlled case series to evaluate MTX infusion in RRD patients. Nevertheless, whether the reattachment rates reported herein in the study eyes were influenced by MTX infusion is uncertain because we did not detect statistical significance in the anatomical parameters tested between the study eyes and the control eyes. In our series, we did not perform electrophysiologic study to assess the toxicity of the dose of methotrexate. The reasons are, firstly, the technical difficulty of performing such test in presence of silicone oil, which is known for its poor electric conductivity, for at least 3 months in most of the patients. Secondly, 41.5% and 34% of patients in the study and control groups, respectively, had established PVR. This represents a significant confounding factor upon interpretation of the electrophysiology results. Nevertheless, we did not detect any of the previously reported complications attributed to MTX use [13]. This finding is of particular importance in corroborating the safety of MTX dose used because the continuous infusion during surgery saturated the ocular tissues with MTX. Furthermore, clearance of the drug was further delayed due to the presence of silicone oil. Both factors would have potentiated the adverse effects of MTX had the dose used been toxic. Another corroborating evidence of the safety of MTX infusion in our series is that 65% of our patients experienced improved vision with a mean improvement of 4 lines. Fifty-five patients (54%) recovered ambulatory vision (≥0.1 Snellen), and 11% had final BCVA of ≥0.4 Snellen. Furthermore, the visual outcome of MTX use in Groups II and III was significantly superior to the respective control groups (*p* = 0.03). An important advantage of MTX infusion is providing stable concentrations of the drug flowing into the ocular tissues. This is compared to the unpredictable therapeutic effect of a single high bolus delivered as intravitreal injection, especially in the presence of intraocular tamponade. The possibility of creation of a depot through saturation of retinal tissues by continuous infusion of MTX and that releases MTX for some time after surgery is interesting and would provide a major advantage over multiple intravitreal injections but yet to be proven by animal studies. Limitations of this study include the discrepant modes of recruiting the study and control groups, and the subsequent inhomogeneity across data in some of the parameters tested, which could be the reason we could not detect the statistical significance of some of the outcomes reported herein.

## 5. Conclusion

Off-label use of intravitreal infusion of MTX during PPV is a safe adjuvant therapy in RRD patients with and without PVR. MTX yields superior functional outcomes in patients at high risk of PVR and patients with no risk of PVR compared to PPV without MTX but not in established PVR cases. PPV with MTX did not confer an additional advantage in terms of retinal reattachment rate compared to PPV without MTX use.

## Figures and Tables

**Table 1:** Baseline characteristics of the study participants.

Group	Gender, *n*		Mean age (years)	Lens status, *n*			Mean follow-up (months)	Mean baseline BCVA^*∗*^
	Male	Female		Phakic	Pseudophakic	Aphakic		
I, *n* = 42	27	15	41	19	21	2	6	0.02
IA, *n* = 30	16	14	46	5	25	0	7	0.02
^*∗∗*^ *P* value	0.35		0.1	0.01			0.003	0.5
II, *n* = 35	23	12	44.5	14	21	0	6	0.07
IIA, *n* = 30	19	11	50	13	17	0	7	0.05
*P* value	0.8		0.1	0.7			≤0.001	0.3
III, *n* = 24	19	5	49	13	11	0	6	0.08
IIIA, *n* = 29	10	19	55	19	10	0	7	0.03
*P* value	0.001		0.1	0.4			0.001	0.5

^*∗*^BCVA, best-corrected visual acuity in Snellen decimal notation; *n*, number. ^*∗∗*^*P* is significant at <0.05.

**Table 2:** MTX use versus anatomical outcome in each subgroup and no MTX use versus anatomical outcome in control.

		Anatomical outcome		^*∗*^ *P* value
		Successful	Recurrent	
Subgroups based on MTX indication	IA	26	4	0.2
	I	31	11	
	IIA	28	2	0.07
	II	27	8	
	IIIA	23	6	0.07
	III	23	1	

MTX, methotrexate. ^*∗*^*P* is significant at <0.05.

**Table 3:** MTX versus mean visual acuity in each subgroup and no MTX use versus mean visual acuity in control.

		Final BCVA					^*∗∗*^ *P* value
		Mean	SD	Median	Minimum	Maximum	
Subgroups based on MTX indication	Control 1	0.04	0.04	0.03	0.001	0.13	0.07
	Established PVR	0.11	0.15	0.05	0.01	0.7	
	Control 2	0.08	0.06	0.1	0.001	0.2	0.03
	High-risk PVR	0.15	0.12	0.16	0.01	0.4	
	Control 3	0.08	0.05	0.1	0.001	0.16	0.03
	No risk of PVR	0.16	0.14	0.16	0.01	0.5	

BCVA, best-corrected visual acuity; MTX, methotrexate; PVR, proliferative vitreoretinopathy; SD, standard deviation. ^*∗∗*^*P* is significant at <0.05.

**Table 4:** Effect of lens status on anatomical outcome in each subgroup and control.

Lens status				Anatomical outcome		^*∗∗*^ *P* value
				Successful	Recurrent	
				count	count	
Subgroups based on MTX indication	Control 1	Lens status	Pseudophakic	23	2	0.1
			Aphakic	0	0	
			Phakic	3	2	
	Established PVR	Lens status	Pseudophakic	14	7	0.3
			Aphakic	1	1	
			Phakic	16	3	
	Control 2	Lens status	Pseudophakic	15	2	0.5
			Aphakic	0	0	
			Phakic	13	0	
	High-risk PVR	Lens status	Pseudophakic	16	5	1
			Aphakic	0	0	
			Phakic	11	3	
	Control 3	Lens status	Pseudophakic	7	3	0.6
			Aphakic	0	0	
			Phakic	16	3	
	No risk of PVR	Lens status	Pseudophakic	10	1	0.4
			Aphakic	0	0	
			Phakic	13	0	

MTX, methotrexate; PVR, proliferative vitreoretinopathy. ^*∗∗*^*P* is significant at <0.05.

**Table 5:** Effect of number of recurrences on anatomical outcome and mean visual acuity in each subgroup and control.

Anatomical outcome						^*∗∗*^ *P* value
				Successful	Recurrent	
Subgroups based on MTX indication	Control 1	Number of recurrences	0	3	0	0.4
			1	9	1	
			2	8	3	
			3	3	0	
			4	3	0	
	Established PVR	Number of recurrences	0	9	4	0.7
			1	14	3	
			2	5	2	
			3	2	2	
			4	1	0	
	Control 2	Number of recurrences	0	28	2	—
	High-risk PVR	Number of recurrences	0	26	7	0.4
			1	1	1	
	Control 3	Number of recurrences	0	23	6	—
	No risk of PVR	Number of recurrences	0	23	1	—

MTX, methotrexate; PVR, proliferative vitreoretinopathy. ^*∗∗*^*P* is significant at <0.05.

**Table 6:** Review of studies on PPV for proliferative vitreoretinopathy complicating rhegmatogenous retinal detachment.

Author	PVR grade	Surgical technique	No. of eyes	Retinal reattachment (%)	Final BCVA
Lewis et al. [10]	23% *C*1-*C*3	91.3% PPV + 14% C3F8	81	81% (single surgery)	85% ≥ 5/200
	77% *D*1-*D*3	8.6% PPV + silicone oil		90% (additional surgeries)	

Lewis and Aaberg [11]	19% *C*3	78% PPV + C3F8	37	68% (single surgery)	59% ≥ 5/200
	81% *D*1-*D*3	22% PPV + silicone oil		73% (additional surgeries)	
				13% (attachment posterior to scleral buckle)	

Silicone Study [12]	PVR C or higher	PPV + C3F8/silicone oil	131 (no prior PPV)	68.5%	44% ≥ 5/200
			134 (prior PPV)	67%	35.5% ≥ 5/200

Asaria et al. [18]	At high risk of PVR	PPV + SF6/C3F8/silicone oil	87 (placebo arm)	71.2%	Stable 12.6%
					Better 45.9%
					Worse 41.3%

Charteris et al. [27]	PVR C	PPV + silicone oil	78 (placebo arm)	51% (single surgery)	∼2 lines gain

Grigoropoulos et al. [26]	PVR C	PPV + C3F8/silicone oil	304	51% (single surgery)	Stable 24%
				72% (additional surgeries)	Better 45%
					Worse 29%

Wickham et al. [29]	86% No PVR	PPV + SF6/C3F8/silicone oil	288 (placebo arm)	86.8% (single surgery)	—

De silva et al. [9]	PVR C	6% PPV + C3F8	145	68%	76% improved or stable
		94% PPV + silicone oil			

Lam et al. [28]	PVR C	PPV + silicone oil	147	81.6%	∼3 lines gain

Current study, 2020	41.5% PVR C	9% PPV + C3F8	42 (PVR C)	74% (single surgery)	4 lines mean gain 54% ≥ 0.1, 11% ≥0.4
	35% high risk of PVR	91% PPV + silicone oil	35 (high risk of PVR)	77% (single surgery)	
	24% no risk of PVR		24 (no risk of PVR)	96% (single surgery)	

BCVA, best-corrected visual acuity; C3F8, octafluoropropane; No., number; PPV, pars plana vitrectomy; PVR, proliferative vitreoretinopathy; SF6, sulfurhexafluoride.

**Table 7:** Review of studies on PPV and adjuvant methotrexate for proliferative vitreoretinopathy complicating rhegmatogenous retinal detachment.

Author	PVR grade	Surgical technique	No. of eyes	Retinal reattachment (%)	Final BCVA
Sadaka et al. [7]	PVR C	PPV + MTX infusion + SF6/C3F8/silicone oil	29	90% (single surgery)	66% ≥ 20/200

Benner et al. [8]	PVR C	PPV + extended PFCL tamponade + 5 bi-weekly MTX injections (100–200 *μ*g/0.05 mL)	5	100%	80% > 20/200

Nourinia et al. [16]	PVR C	PPV + intra-silicone oil injection of MTX 250 *μ*g (3 injections, 3-week interval)	11	82% total reattachment	∼6 lines gain
				18% reattachment posterior to the equator	
				Silicone oil was not removed in 82% of patients	

Falavarjani et al. [17]	PVR C	PPV + single intrasilicone oil injection of MTX 250 *μ*g	22 (treatment arm)	95.5%	No statistically significant difference between groups
			22 (control arm)	77.3%	

Current study, 2020	41.5% PVR C	PPV + MTX infusion + C3F8/silicone oil	42 (PVR C)	74% (single surgery)	4 lines mean gain 54% ≥ 0.1, 11% ≥ 0.4
	35% high risk of PVR		35 (high risk of PVR)	77% (single surgery)	
			24 (no risk of PVR)	96% (single surgery)	
	24% no risk of PVR				

BCVA, best-corrected visual acuity; C3F8, octafluoropropane; *μ*g, microgram; mL, milliliter; MTX, methotrexate; No., number; PFCL, perfluorocarbon liquid; PPV, pars plana vitrectomy; PVR, proliferative vitreoretinopathy; SF6, sulfurhexafluoride.

## Data Availability

The data collected from history taking and clinical examination of patients recruited in this study are confidential. Access to these data is restricted by El Baha Eye Center in accordance with patients' data protection policy. Data are available for researchers who meet the criteria for access to confidential data by contacting the lead investigator.
